# Epidemiological and Evolutionary Analysis of West Nile Virus Lineage 2 in Italy

**DOI:** 10.3390/v15010035

**Published:** 2022-12-22

**Authors:** Giulia Mencattelli, Andrea Silverj, Federica Iapaolo, Carla Ippoliti, Liana Teodori, Annapia Di Gennaro, Valentina Curini, Luca Candeloro, Annamaria Conte, Andrea Polci, Daniela Morelli, Maria Gabriella Perrotta, Giovanni Marini, Roberto Rosà, Federica Monaco, Nicola Segata, Annapaola Rizzoli, Omar Rota-Stabelli, Giovanni Savini, West Nile Working Group

**Affiliations:** 1 Istituto Zooprofilattico Sperimentale dell’Abruzzo e del Molise, 64100 Teramo, Italy; 2 Centre Agriculture Food Environment, University of Trento, 38010 San Michele all’Adige, Italy; 3 Research and Innovation Centre, Fondazione Edmund Mach, 38010 San Michele all’Adige, Italy; 4 Department CIBIO, University of Trento, 38123 Trento, Italy; 5 Centro Nazionale di Lotta ed Emergenza Contro le Malattie Animali, 00161 Rome, Italy

**Keywords:** West Nile virus lineage 2, epidemiology, evolution, early-warning, Italy

## Abstract

West Nile virus (WNV) is a mosquito-borne virus potentially causing serious illness in humans and other animals. Since 2004, several studies have highlighted the progressive spread of WNV Lineage 2 (L2) in Europe, with Italy being one of the countries with the highest number of cases of West Nile disease reported. In this paper, we give an overview of the epidemiological and genetic features characterising the spread and evolution of WNV L2 in Italy, leveraging data obtained from national surveillance activities between 2011 and 2021, including 46 newly assembled genomes that were analysed under both phylogeographic and phylodynamic frameworks. In addition, to better understand the seasonal patterns of the virus, we used a machine learning model predicting areas at high-risk of WNV spread. Our results show a progressive increase in WNV L2 in Italy, clarifying the dynamics of interregional circulation, with no significant introductions from other countries in recent years. Moreover, the predicting model identified the presence of suitable conditions for the 2022 earlier and wider spread of WNV in Italy, underlining the importance of using quantitative models for early warning detection of WNV outbreaks. Taken together, these findings can be used as a reference to develop new strategies to mitigate the impact of the pathogen on human and other animal health in endemic areas and new regions.

## 1. Introduction

West Nile virus (WNV) is a positive strand RNA virus, belonging to the *Flaviviridae* family, genus *Flavivirus*, within the Japanese encephalitis virus serocomplex [1,2]. It is maintained in nature through a transmission cycle involving competent mosquitoes and birds acting as main vectors and major amplifiers, respectively [3,4]. Amongst the group of birds, corvids and raptors are highly susceptible. In these groups, WNV infection can result in severe, sometimes fatal neurological disorders [2,5,6]. In other groups, symptoms are less frequently observed, and in some cases chronic infection may develop [5,7,8,9]. Migratory birds can play a key role in introducing the virus to new regions, while resident competent birds are mostly involved in local transmission, which might eventually lead to the endemic circulation of the virus [5,10,11]. Humans, horses, and other vertebrates, such as reptiles, amphibians, and several mammals, are regarded as WNV incidental dead-end hosts [2,4]. In equids, only the minority of cases have severe clinical signs, associated to neurological symptoms, resulting in substantial economic costs and serious emotional distress [12,13]. In humans, WNV infection is generally asymptomatic (80%) [14]. In 20% of cases, however, it can lead to West Nile fever (WNF), characterised by flu-like symptoms. In a lower percentage of cases, mainly in old or immunocompromised people, infections can also cause severe and even fatal diseases characterised by neurological manifestations [2,15].

WNV was first detected in 1937 in Uganda. It has been circulating outside East Africa since the 1950s [2,3,4,15] and nowadays is reported in many countries worldwide [2,4,16]. Phylogenetic studies have revealed that WNV comprises at least eight lineages. WNV lineage 1 (L1) and lineage 2 (L2) are the most pathogenic and widespread, causing several outbreaks in birds, humans, and horses around the world [17]. From the 1960s to 2004, L1 was the main lineage circulating in Europe [18]. Since its first appearance in Hungary in 2004, L2 quickly spread throughout Europe, becoming endemic in many European countries [19]. Nowadays, it is the most prevalent WNV lineage circulating in the continent [20,21].

In Italy, the first West Nile disease (WND) outbreak was reported in the Tuscany region in 1998. The strain belonged to the WNV L1 genotype and was responsible for severe neurological cases and deaths among horses [22]. 

The virus has been monitored in the country since 2001. The surveillance plan did not detect any relevant circulation until 2005, when WNV antibodies were detected in sentinel chickens in northern Italy (see Material and Methods, as well as sample and data collection sections, for the details of the surveillance plan) [23]. Following the first years in which the surveillance plan was tackled almost separately by the human and veterinary health systems, a One-Health integrated surveillance program has been implemented since 2016. It integrates human, animal, and entomological surveillance to detect seasonal WNV circulation early. The detection of WNV in any host/vector is confirmed by a network of reference laboratories coordinated by the Ministry of Health, the Istituto Superiore di Sanità (National Reference Laboratory for WND in humans), and the Istituto Zooprofilattico of Abruzzo and Molise (IZS-Teramo) (National Reference Laboratory for WND in animals) (https://westnile.izs.it/j6_wnd/home, https://www.epicentro.iss.it/westnile/, accessed on 15 November 2022). The surveillance activities are refined every year according to the new WNV epidemiological findings in the country.

In 2008, several WNV L1 cases were reported in Emilia-Romagna, Veneto, and Lombardy [24]. In the following years, the virus became endemic in the northern part of the country and between 2010 and 2011 spread to the south [15]. Evidence of WNV L2 circulation in Italy was first reported in 2011 [25,26]. After its first appearance, it rapidly spread all over the country replacing WNV L1, which was only sporadically detected until October 2020, when it re-emerged in Italy in the Campania region [18]. The current 2022 vector season is characterised by an intense co-circulation of WNV L1 and L2 virulent strains, detected at the same time in mosquitoes, birds, horses, and humans [27], as reported in the Annual Epidemiological bulletins produced for Italy by IZS-Teramo (https://westnile.izs.it/j6_wnd/periodicalItaly, accessed on 10 November 2022).

An exceptional number of WNV L2 cases were observed in Italy between June and November 2018 [21], with 577 human cases confirmed, including 230 neuroinvasive diseases (39.86%), 279 fevers (48.35%), and 42 deaths (7.27%). They occurred in Veneto, Emilia-Romagna, Lombardy, Piedmont, Sardinia, Friuli-Venezia Giulia, and Molise (only 1 case imported from Greece) regions. An abnormal number of WND cases were also observed in horses, birds, and mosquitoes, with 238 cases reported in equids (IgM or molecular test), while WNV L2 RNA was detected in 239 resident target birds (*Pica*, *Corvus corone cornix*, and *Garrulus glandarius*), 109 wild birds, and 433 mosquito pools in the Emilia-Romagna, Lombardy, Piedmont, Friuli-Venezia Giulia, Veneto, Sardinia, Lazio, Basilicata, and Puglia regions (https://storymaps.arcgis.com/collections/b50666024702441dac792d0cb3aee32c, year 2018, accessed on 10 November 2022). Currently, Italy is the European country with the highest number of WNV cases, and most of them are associated with WNV L2 infections (see Italian Epidemiological reports https://westnile.izs.it/j6_wnd/home, accessed on 10 November 2022). 

In this paper, we reconstructed the phylogeny, phylogeography, and phylodynamics of WNV L2 in Italy to better characterise its spread and evolution. The epidemiological trend of WNV L2, since its first appearance in the country in 2011, is also described by analysing the seasonal patterns and the viral prevalence in mosquitoes, birds, horses, and humans over the years. These analyses gave us the opportunity to assess the accuracy of the recent early warning system based on the environmental and climate model [28] in predicting WND epidemic behaviour during the 2021 and 2022 vector seasons. 

## 2. Materials and Methods

### 2.1. Sample and Data Collection

In 2001, following the first outbreaks of WNV L1 reported in Tuscany in the late ‘90s, a WNV and Usutu virus integrated surveillance plan was implemented in Italy. At that time, the plan aimed to detect the introduction and local spread of these viruses. Fifteen WNV-at-risk areas were selected according to their suitable eco-climatic conditions and monitored by using an approach based on serological screening in sentinel animals (horses and poultry), wild bird mortality, and mosquito surveillance. In 2002, hospitalised human cases of fever with rash or encephalitis and meningitis recorded in regions where virus circulation was evidenced by the animal surveillance, started being reported. No WNV L2 cases were detected until 2011, when the lineage first appeared in northern regions, soon becoming endemic. Since this year, WNV L2 samples were collected under an integrated surveillance on birds, mosquitoes, and humans, activated on a regional scale, in northern regions [29], while WNV monitoring in the rest of Italy was based on serological screening in sentinel horses and chickens. Since this year, samples were collected.

In 2016, the first national plan integrating human, animal (equids, resident, and wild birds), and mosquito surveillance was issued. Its evolution in the following years led to the 2020–2025 Integrated Surveillance and Response Plan for Arboviruses (PNA), which is currently still in place (https://westnile.izs.it/j6_wnd/ministeriale, accessed on 10 November 2022). Seasonal surveillance activities are defined on the basis of the previous year’s virus circulation (risk areas), while any WNV detection in birds, mosquitoes, equids, and humans triggers the activation of prevention measures (blood and transplant measures including nucleic acid testing, vector control, and risk communication campaigns aimed at citizens). Therefore, the start date of these measures can vary each year and, in each province, depending on the viral circulation. 

Any positive results from local surveillance activities are confirmed by the National Reference Centre for Foreign Animal Diseases (CESME) at IZS-Teramo. Notifications of outbreaks are registered by the official veterinary authorities in the national information system for the notification of outbreaks in animals (SIMAN) [30]. The notification system is designed to register and document the occurrence and evolution of important infectious animal diseases according to Council Directive 82/894/EC [29]. 

### 2.2. Sample Analysis

#### Tissue Homogenisation, RNA Extraction, and Real-Time RT-PCR

At IZS-Teramo, bird tissue samples (heart, kidney, spleen, and brain) and pools of mosquitoes were homogenised in phosphate-buffered saline (PBS) with antibiotics. WNV RNA was extracted by using the MagMAX CORE Nucleic Acid Purification KIT (Applied Biosystem, Thermo Fisher Scientific, Life Technologies Corporation, Waltham, MA, USA), according to the manufacturer’s instructions. Every extracted RNA was subjected to one-step quantitative reverse transcription polymerase chain reactions (qRT-PCR) to detect WNV-L1 and/or -L2, and all known lineages of WNV by using the Superscript III Platinum OneStep qRT-PCR System (Invitrogen, Thermo Fisher Scientific, Life Technologies Corporation, Waltham, MA, USA) [31]. 

### 2.3. Epidemiological Data Analysis

All data collected within the Italian WNV surveillance plan between 2011 and 2021 (see Table S1) have been processed using LibreOffice Calc v 365 [32] and Microsoft Excel v 4.2.2 [33]. Data collected between 2011 and 2021 were analysed with LibreOffice Calc, Microsoft Excel, and R version 4.1.2 [34].

### 2.4. Sequence Data Preparation and Retrieval

#### 2.4.1. Whole Genome Sequencing, Genome Assembly, and Sequence Processing at NCBI

**Figure 1:**
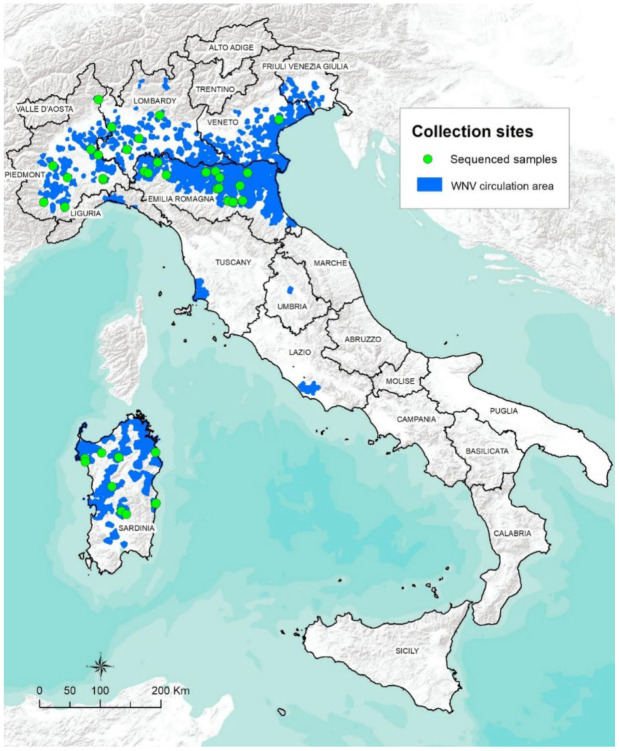
Geo-localization of WNV L2 sequenced sample collection sites. In blue, the WNV L2 circulation municipalities obtained from veterinary sample collection performed between 2011 and 2021 are displayed. The collection sites of samples from which sequences were obtained are shown in green.

Purified nucleic acids were sequenced by Next Generation Sequencing, as described in detail in [35]. WNV L2 consensus sequences were obtained using iVar v 1.3.1 [36] after reads were mapped to various WNV L2 reference sequences (KU573082_Italy_2013, MN652880_Greece_2018, KT207792_Italy_2014, KP789954_Italy_2014, and HQ537483_Greece_2010) by using Snippy (https://github.com/tseemann/snippy, accessed on 1 November 2022). A map showing the geo-localisation of the collection sites of the sequenced samples can be found in Figure 1.

#### 2.4.2. Download of Reference Genomes

All available WNV sequences ≥ 200 nt have been downloaded from NCBI by using a custom R script for automatic sequence retrieval, which allows one to select sequences of the desired length by leveraging the “rentrez” R package (https://cran.r-project.org/web/packages/rentrez/index.html, accessed on 25 October 2022).

#### 2.4.3. Sequence Metadata Collection and Curation

Metadata for the newly generated sequences were obtained from the Laboratory Information Managements Systems (SILAB) at IZS-Teramo. SILAB is a web application designed by IZS-Teramo IT staff that uses an automated electronic recording function to support all diagnostic activities performed on the incoming samples, from their registration to the report release (https://www.izs.it/IZS/Engine/RAServePG.php/P/257610010719/L/1, accessed on 20 October 2022). All the information recorded in SILAB is originally collected and presented in the Vetinfo portal (https://www.vetinfo.it/, accessed on 20 October 2022) and subsequently reported in the PNA (https://www.vetinfo.it/, https://www.salute.gov.it/imgs/C_17_pubblicazioni_2947_allegato.pdf, accessed on 20 October 2022). 

Metadata of all WNV sequences present in NCBI were downloaded from the NCBI virus platform (https://www.ncbi.nlm.nih.gov/labs/virus/vssi/#/, accessed on 20 October 2022) as a single csv file, including all available fields.

#### 2.4.4. Sequence Data Cleaning and Formatting

Only data for sequences longer than 10 kb (i.e., sequences covering almost the entire WNV genome) were retained for subsequent steps. All sequences were filtered for quality, converting all unusual nucleotides into “N” letters, and then sequences which contained a percentage of ambiguous bases superior to 10% were removed. After this step, a total of 2478 genomes, 95 of which came from Italy, were selected for further analysis. For these sequences, we checked the cases lacking in information regarding the collection date and host, attempting to retrieve it from the corresponding paper, if available. When unable to identify the collection date, we put a putative date, if at least the year was known, choosing 15/06 (i.e., approximately the half of the year). We filled in all fields lacking in data with “NA” values, obtaining a resulting table of curated metadata (Table S2). All sequences were annotated using custom Python scripts and the newly generated tables with curated metadata, formatting the headers in a “>id|country|yyyy/mm/dd” format.

### 2.5. Phylogenetic Analysis 

#### 2.5.1. Subsampling for Molecular Clock Analysis

In order to reduce redundancy and make data suitable for molecular clock analysis, we carried out a subsampling of our main dataset. First, a maximum-likelihood phylogeny using FastTreev2.1.10 [37] (as specified in the next sections of this study) was reconstructed. As all American genomes (representing 83.9% of our dataset, 2079 sequences out of 2478) belonged to the same clade and were part of the L1 lineage, a subsampling of these sequences was carried out as well. Genome-sampler [38] was used to perform our selection. A total of 49 selected sequences resulted by sampling according to collection date (with a 1095-day sampling frequency) and viral diversity (50%). These sequences were then merged with the remaining quality-checked genomes from all possible world locations, and the sampling step was repeated with the same tool by using them as context sequences (defined as genomes obtained from a global community resource), while using our selection of Italian genomes as focal sequences (i.e., genomes obtained locally). We sampled every 90 days, with a diversity threshold of 0.95 and sampling near neighbours of the focal sequences at 0.99 percent genome diversity, picking up 3 sequences per cluster. A final dataset of 370 sequences (95 genomes from Italy and 275 from 43 other countries) was generated and used for further analysis. 

#### 2.5.2. Alignment and Recombination Detection

All the datasets were aligned individually using MAFFTv7.490 [39], with the “--auto” option. All the datasets, excluding the one comprising all WNV L2 Italian sequences (very similar to each other), were trimmed using trimAlv1.2 [40] with the option “-automated1”. The presence of recombinant sequences in the final dataset was checked by running the RDP4 program [41], with default options. This program uses phylogenetic-based (BootScan, RDP, Siscan) and substitution-based (GenConv, Maxchi, Chimaera, 3SEQ) methods to infer recombination events. A recombination event was regarded as true when detected by at least 5 methods out of 7. The sequences EF429199.1 from South Africa and GQ851604.1 from India were excluded from our final set, as they were indicated as suspected recombinants. The dataset without recombinant sequences (*n* = 368) was realigned and trimmed using the same methods. 

#### 2.5.3. Model Selection

Model selection was carried out on all datasets using Modelfinder [42], implemented in IQTREEv2.1.2 [43], using parameters “-T AUTO -m TESTONLY”. The best-fit model for both the worldwide and the Bayesian down-sampled datasets was GTR + F + I + G4, chosen according to both Akaike information criterion (AIC) and Bayesian information criterion (BIC). 

#### 2.5.4. Maximum-Likelihood Phylogenies

To have an initial estimation of the phylogenetic signal in our dataset, FastTree 2 [35] was used to reconstruct a worldwide phylogeny of all WNV sequences (*n* = 2478) that passed our quality control, with the command “FastTreeDbl -nt -gtr -gamma -log logfile -pseudo”. A maximum likelihood phylogeny of the subset dataset (*n* = 368) was reconstructed by using RAxMLv8.2.12 [44], with commands “-p 1989 -m GTRGAMMAI -x 2483 -# 100 -f a -T 20”. Clades were annotated using the resulting topology when having bootstrap supports ≥90%.

#### 2.5.5. Molecular Clock

BEASTv2.7 [45] was used to obtain phylogenies of the sequences using different sets of priors and models (Table S3) to explore their effect and influence on our phylogenetic reconstruction. Our subsampled dataset of 368 genomes was employed to reconstruct the overall genome evolution of WNV. The topologies of all trees obtained were compared together and with the maximum-likelihood tree to test the robustness of the reconstruction.

#### 2.5.6. Phylogeographic and Phylodynamic Analysis of the Italian Clade of WNV L2

A clade comprising almost all Italian sequences of WNV was found in each tree, with bootstrap = 100 and posterior probability = 1. The sequences belonging to this Italian clade (*n* = 74) were re-annotated, including the name of the specific region of origin and, for those lacking an exact date, a refined estimation of the collection date based on the median of the collection dates of the sequences with complete metadata. The previous steps (alignment, trimming, model selection and tree building with RAxML and BEAST2) were therefore repeated. Phylogeography was reconstructed by using continuous traits (latitudinal and longitudinal coordinates for each sequence) in BEASTv1.10.4 [46]. When the exact position was not available, we approximated the location using the coordinates of the municipality from which the sample was collected (for detailed information about co-ordinates please contact the authors). We divided the analysis into two different partitions (one for sequence data and the other for continuous coordinates) and used the Cauchy RRW substitution model for our location partition, with bivariate traits representing latitude and longitude, adding random jitter to the tips (jitter window size: 0.01). For the location partition, we selected the option to reconstruct states for all ancestors. We employed an uncorrelated relaxed clock with a log-normal distribution and a coalescent Bayesian skyline tree prior. We set a MCMC length of 500 × 106 generations, sampling every 50,000 steps. Convergence was assessed using Tracerv1.7.1 [47], ensuring that all parameters were above a significant threshold of ESS (>200). In parallel, a phylogeographic reconstruction using discrete characters was carried out using BEAST2 for the same dataset and under the same set of parameters (excluding the modelling of the character state), obtaining posterior probability estimates for each location at each node. To characterise the dynamic of the viral population in Italy, coalescent Bayesian skyline and birth–death skyline serial analyses were performed using different sets of models and parameters implemented in BEAST2 (see Table S3). The birth–death skyline serial model made it possible to estimate the effective reproductive number (Re), which is the average number of secondary infections caused by an infected individual at a given time during the epidemic. 

### 2.6. Ecological and Epidemiological Modelling

In [28], the authors presented a model based on climatic and environmental factors (daytime and nighttime land surface temperature, normalised difference vegetation index, and surface soil moisture) that produces, two weeks in advance, risk maps for WNV circulation throughout Italy using a decision-tree-based ensemble machine learning algorithm (XGBoost, https://xgboost.readthedocs.io/en/latest/build.html, accessed on 20 October 2022).

This model, initially based on 2017–2019 data, was later updated and calibrated using the additional 2020 epidemic data and here applied for a comparison between risk maps in 2021 and 2022. 

## 3. Results

### 3.1. Epidemiological Scenario

**Figure 2:**
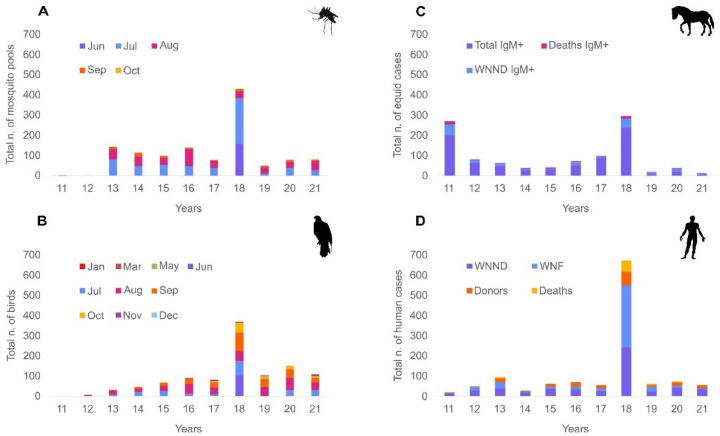
Total yearly number (from 2011 to 2021) of: (**A**) WNV L2 rt-PCR positive mosquito pools; (**B**) WNV L2 rt-PCR positive wild and target birds; (**C**) Total WNV IgM+ cases, IgM+ West Nile neuroinvasive disease (WNND), and IgM+ deaths among equids, not lineage specific; (**D**) Human WNND, West Nile fever (WNF), donors, deaths, and molecular and/or serological positive tests. For (**A**,**B**), the temporal distribution is shown per month and year, with cases reported in the months of: January (Jan); March (Mar); May; June (Jun); July (Jul); August (Aug); September (Sep); October (Oct); November (Nov); and December (Dec). Different colours representing each month are displayed in the legend. In (**C**,**D**), the temporal distribution is shown per year.

Based on all the data collected by the national surveillance plan between 2011 and 2021 in Italy (see Table S1), WNV cases recorded in mosquito pools, birds, horses, and humans were mapped as shown in Figure 2.

WNV L2 typically circulates in the environment among birds and mosquitoes between July and October, with peak activities shown in August (mosquitoes, birds) and September (birds). In mosquito vectors, an increased viral transmission was observed in 2013, 2016, and 2018, a tendency also confirmed by human data (not lineage-specific). The 2018 epidemic season was characterised by a strong recrudescence in mosquitoes, birds, horses, and human cases, with earlier and higher incidence of infections already registered in the month of June. In recent years (2016–2021), an increased viral transmission in birds was observed in November. Serological studies (not lineage-specific) conducted among equids highlighted an intense viral circulation in these animals in 2011.

### 3.2. Genetic Scenario

#### 3.2.1. Genome Sequence Analysis

Illumina sequencing produced an average total number of raw reads per sample of about 1,647,235, and the average total numbers of trimmed reads is 1,618,180. The numbers of mapped reads (151 nucleotides [nt] in length) ranged from 252,431 to 289,804, with coverage depth ranging from 1267× to 6858×. Overall, consensus sequences were characterised by complete WNV L2 whole genomes. The 45 WNV L2 whole genome sequences obtained at the CESME were uploaded to BankIT NCBI (https://submit.ncbi.nlm.nih.gov/about/bankit/, accessed on 20 October 2022) in May 2021 and March 2022.

#### 3.2.2. World Scale Phylogenomics of WNV

**Figure 3:**
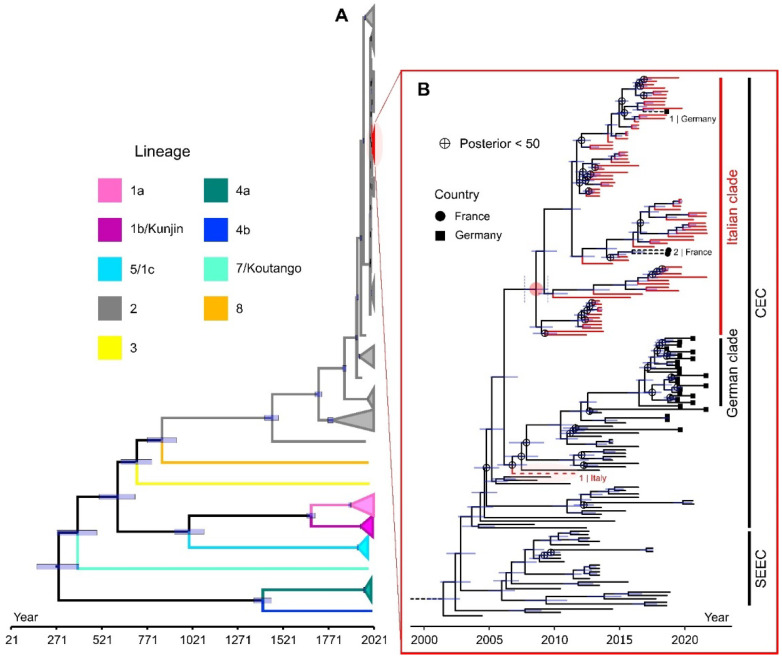
(**A**) Worldwide time tree of 368 WNV genome sequences from 44 different countries. WNV lineages are indicated with different colours. Uncertain dates are represented with blue bars showing the 95% HPDs. Nodes defining the major lineages have all posteriors > 70 (in most cases > 90). A clade which includes the majority of the WNV L2 sequences from Italy is highlighted in red, inside the general WNV L2 subtree (in grey). (**B**) A magnification of the tree, showing the position of the Italian clade of WNV L2 among the central European clade (CEC). A WNV L2 genome sampled in Italy (JN858070.1, sampled in 2011 in the city of Ancona) and not belonging to the identified clade is shown with a red dotted line. The south-eastern European clade (SEEC), sister of the CEC, is also shown in the tree. Nodes with posterior probability < 50 are indicated with a circled cross.

Phylogenetic reconstructions for the worldwide dataset comprising 368 sequences were similar across maximum-likelihood and Bayesian methods. For Bayesian trees, evolutionary rates and divergence times were different according to the specific combination of models used (see Table S3 and Figure S1). The majority of WNV L2 Italian genomes (74 out of a total of 95 included in the analysis) clustered together in a large clade (Figure 3, highlighted in red), which was retrieved with high support in each tree that was reconstructed, independently of the framework and of the models employed (see Table S3).

This group also included two sequences from France (MT863560.1, MT863561.1) and one from Germany (MH910045.1), both from the year 2018. The genomes from France clustered with sequences mostly from Piedmont, while the German sequence was part of a group of sequences from Veneto and Sardinia. Only one Italian sequence (JN858070.1, sampled in 2011 in the city of Ancona) of WNV L2 was not included in the clade, forming a poorly supported group with the German clade of the virus and other sequences from Austria and Slovakia (Figure 3B).

#### 3.2.3. Phylodynamic and Phylogeographic Analysis of the WNV L2 Italian Clade

**Figure 4:**
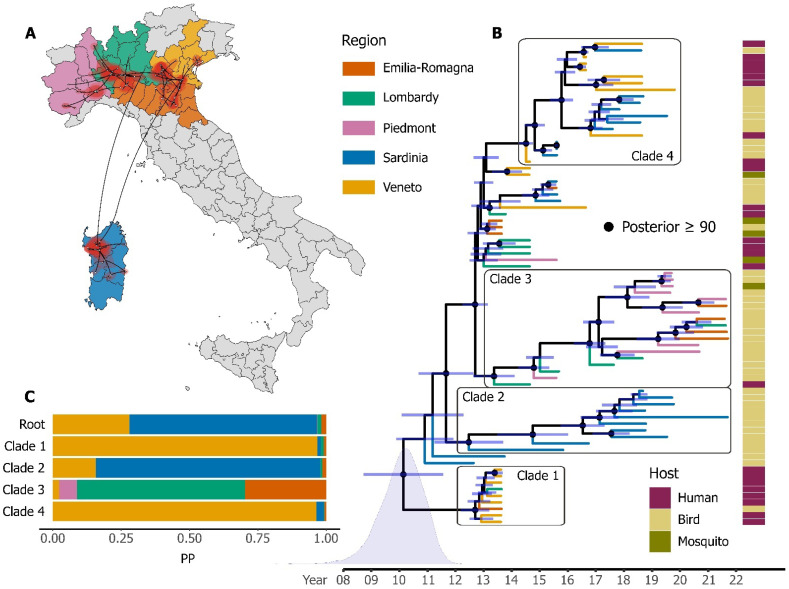
Phylogeographic analysis of WNV L2 sequences, part of the identified Italian clade. Colours indicate samples originating from five different Italian regions where the virus was detected. (**A**) The geographic position and the connection between all genomes analysed are shown. (**B**) Uncertain dates are represented with light blue bars showing the 95% HPDs. Nodes with high posterior probabilities (≥90) are indicated with black dots. The host from which the sample was reconstructed is indicated in a column on the right with bars of different colours. The blue curve at the root represents the posterior density. (**C**) A table showing posterior probabilities for the location state of the root and the four highly supported clades.

Phylogenies built for the identified monophyletic Italian clade were consistent among methods and models, always showing the presence of the same four main well supported clades (Figure 4). 

Clade 1, at the base of the tree, contained variants that were not sampled anymore in the following years. A group of sequences only from Sardinia defines clade 2. Clade 3 included sequences from northern Italian regions (Piedmont, Emilia-Romagna, and Lombardy), while clade 4 had genomes from Veneto and Sardinia. Some sequences cannot be placed steadily in a group (their position changes among different trees) and come from different regions. The diffusion of the virus in the early years seemed to have started from two main areas (Sardinia and Veneto; Figures S2 and S3, Videos S1 and S2), moving further towards nearby regions in the following seasons and bursting in 2018 with an unusually large epidemic. The state of the character at the root suggests that the last common ancestor of these sequences was in Sardinia (posterior probability = 68.59), even though the signal is not highly supported and competes with the one from the Veneto region (posterior probability = 27.9). The ancestors of the other clades have a clearer geographic location (Veneto for clade 1 and 4, Sardinia for clade 2, Emilia-Romagna for clade 3).

**Figure 5:**
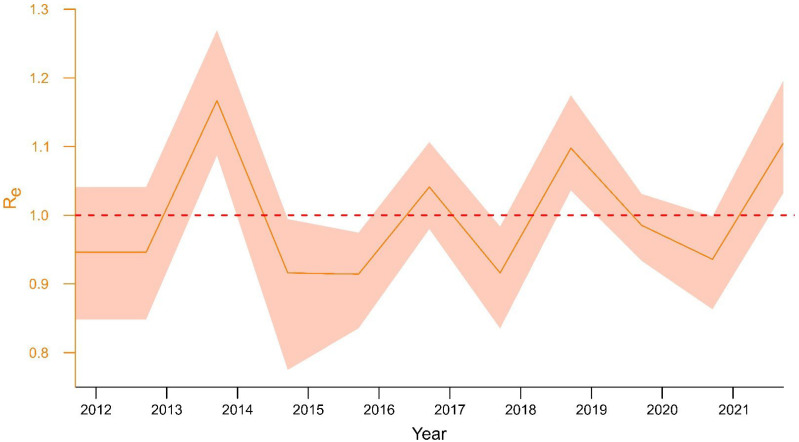
Birth–death skyline serial analysis of WNV L2 sequences of the identified Italian clade. Reproductive number (Re) is plotted for a timespan that goes from 2012 to 2021. (Re) > 1 indicates a growth of the epidemic.

Bayesian skyline plot analysis was used to investigate population dynamics of WNV L2 circulating in Italy. Plots reconstructed under both coalescent (Figure S4) and birth–death models (Figure 5) gave similar results, indicating an increase in the circulation of the virus for the 2013 and 2018 epidemic seasons. 

A more modest growth is observed in 2016 and 2021, whilst a decline is shown for all the other years. The estimates of the Re were in line with this scenario (Figure 5). The peaks of 2013 and 2018 are preceded by a phase of some months in which population growth seems to increase constantly.

### 3.3. Epidemiological and Ecological Modelling

**Figure 6:**
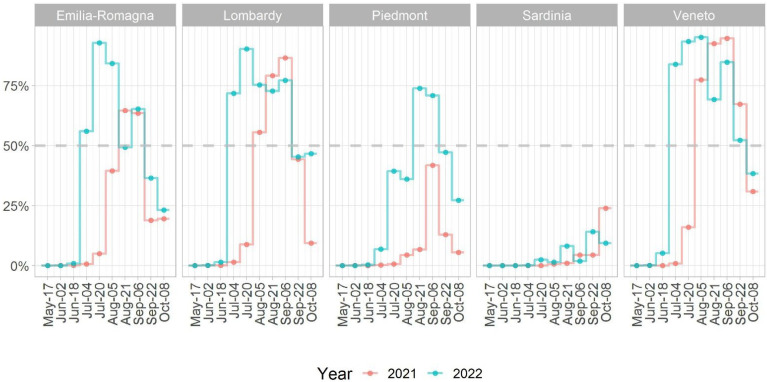
Median values of WNV circulation suitability (probability ranging from 0 to 100%) in five Italian regions during the epidemic season in 2021 (orange line) and 2022 (light blue line). The spatial distribution of the probability across Italy is accessible through the web application https://mapserver.izs.it/gis_wn_predictions/, accessed on 20 October 2022, in which it is possible to view the risk map for WNV throughout the Italian territory with forecast periods of 16 days.

The eco-climatic model developed by Candeloro et al. [28] confirms the presence of suitable conditions for WNV L2 2022 early spread. Compared to 2021, the 2022 epidemic season is characterised by a higher probability of WNV circulation and by an earlier start of the vector season (1–1.5 months). The most endangered regions are considered Emilia-Romagna, Lombardy, and Veneto, where the median values of WNV circulation suitability range from 0.56 to 0.84 already at the end of June. Results are shown in Figure 6.

According to these predictions, preliminary data analysis performed on notified outbreaks in SIMAN occurred in the 2022 epidemic season, which confirmed an earlier and wider WNV circulation in Italy compared to 2021.

## 4. Discussion

Italy is one of the European countries reporting the highest numbers of human and animal WND cases. WNV L1 constantly circulated in the area from 2008 until 2011. Since 2012, the epidemiological scenario has been primarily dominated by WNV L2—at least until 2022, when L1 and L2 WNV strains surprisingly started co-circulating again, with numerous infections and co-infections reported in humans, horses, birds, and mosquitoes [27], (https://westnile.izs.it/j6_wnd/periodicalItalyDocs?docYear=2022, accessed on 20 October 2022).

Since its first appearance, WNV L2 has been reported every year in Italy. Monitoring activities detected WNV L2 infections in 10 out of 20 Italian regions (Emilia-Romagna, Lombardy, Piedmont, Liguria, Veneto, Friuli-Venezia Giulia, Umbria, Tuscany, Lazio, and Sardinia), as well as IgM positive horses in 12 regions (Emilia-Romagna, Friuli-Venezia Giulia, Veneto, Piedmont, Sardinia, Lombardy, Tuscany, Lazio, Basilicata, Puglia, Calabria, and Sicily), evidencing a wide viral diffusion all over the country. Northern Italy and Sardinia stand out as the most affected areas and are nowadays considered endemic (Figure 1), (IZS-Teramo Annual Epidemiological Bulletins, https://westnile.izs.it/j6_wnd/wndItalia, https://westnile.izs.it/j6_wnd/wndItaliaPeriodici, accessed on 20 October 2022). According to the data, WNV L2 typically circulates between July and October in Italy, with peaks observed in the months of August and September. However, variations in WNV transmission patterns are not unusual in the country: (i) the 2018 and 2022 epidemic seasons were characterised by an earlier and increased incidence of transmission, with infections detected as early as June [27], (IZS-Teramo Annual Epidemiological Bulletins, Figure 2); (ii) in the last years (2016–2021), an increasing number of cases have been reported in November, showing a right shift in WND epidemiological curve’s tail-end (Results, Figure 2); (iii) WNV-L2 strains have been detected among birds in December and January (2017, 2019, 2022), even in low-risk areas (i.e., Umbria region—2019, 2022) (IZS-Teramo Annual Epidemiological Bulletins) [9,35]; and (iv) an elevated number of WND cases (serological findings, not lineage specific) were observed in horses in 2011, likely due to WNV L1 infection, the most prevalent lineage circulating in Italy in that year [29], or vaccine seroconversion.

Differences in the WNV transmission patterns in the WND epidemics also emerged clearly from the investigation on the population dynamics of the WNV L2 clade inferred using Bayesian skyline plots, under both a coalescent (Figure S4) and a birth–death model (Figure 5). The results derived from this investigation in fact highlighted, in consistency with the observed surveillance activity data, the increased viral transmission which occurred during the 2013, 2016, and 2018 epidemic seasons (IZS-Teramo Annual Epidemiological Bulletins). According to the Bayesian skyline plots, in case of favourable events (e.g., temperature and humidity), the vector and viral population abnormal growth might be evident before summer, well ahead of the epidemic season.

All reconstructed trees gave comparable results despite the method used. All confirmed the overall scenario described in previous studies, in which different lineages of WNV were defined [17]. As revealed by the molecular clocks and phylogeographic analysis, the first introduction of L2 strains in the Italian territory likely occurred between 2008 and 2011 in Sardinia or Veneto (Figure 4; Figures S2 and S3; Supplementary Videos S1 and S2). In line with other studies [48], in all our phylogenetic reconstructions WNV L2 Italian genomes tended to cluster together within the Central and Eastern European clades (Figure 3), excluding any recent external introduction of the virus, but at the same time confirming the existence of an endemicity status of WNV L2 in Italy. Viral endemicity is most probably assured by the establishment of an endemic cycle through resident birds and vector competent mosquitoes and by overwintering strategies put in place by the virus to survive the winter season [35,49,50].

Phylogenetic analysis indicated the presence of a main WNV L2 Italian group, which was highly supported across all our different phylogenies. Our phylogeographic analysis and ancestral character state reconstruction suggest that the origin of the sequences included in this group was most likely in Sardinia, with a posterior probability of 68.59 for this region as ancestral location. This is an interesting possible scenario that had not been suggested by previous studies, but which could be the result of a bias due to our sequence dataset, which only includes data for complete genomes and excludes the partial genome sequences obtained in northeast Italy in 2011 [26]. As described by previous studies, the most probable scenario is a southward expansion of the WNV L2 from Central European countries [19,51] to north-eastern regions of Italy—possibly to the Veneto region (for which the support in our ancestral character state reconstruction is 27.9), later expanding to other regions [26,51], with a genetic flow probably sustained by birds migrating either along the south-eastern migration route from Europe and western Asia to Africa, or along short migration routes from Central to Southern Europe [26]. Our molecular clock displayed in Figure 2 also suggests this to be the most likely scenario, as the sister group and other closely related genomes of the Italian L2 clade are from central European countries (Austria, Germany, Slovakia, Czech Republic, Serbia, and Hungary), as can be checked by looking at the full tree used to generate Figure 2 (see the file “wnv_world368_clock_trimmed_tipd_gtr_gamma4_strict_cc.tre” in Data Availability Statement section, https://figshare.com/articles/dataset/West_Nile_virus_lineage_2_tree_files/21518541/1, accessed on 20 October 2022). Further studies are required to clarify the first introductory events of this lineage in Italy. 

Our phylogeographic analysis shows four main Italian groups that were highly supported, regardless of the framework (maximum-likelihood or Bayesian) and on the models used: (i) clade 1, characterised by sequences mostly from Veneto that became extinct in 2013–2014, as previously described in the literature [20]; (ii) clade 2, including only Sardinian strains, which probably arrived in Italy via infected birds from neighbouring countries [52] and then possibly spread to north-western Italy, indicating a possible local variant from Sardinia; (iii) clade 3, with genomes circulating in Emilia-Romagna and Piedmont, with a few sequences from Lombardy; and iv) clade 4, including mostly genomes from Veneto and Sardinia. The group division is mostly related to the diverse regions of Italy, as also presented in the results (Figure 4), suggesting initial viral interregional circulation followed by a local establishment and adaptation to diverse eco-climatic conditions and maintenance hosts, which lead to strain genetic diversity and constant intra-regional WNV L2 circulation.

These findings underline the importance of the national surveillance plan and the urgent need for the development of mathematical models able to predict, early on, the WNV behaviour in the following vector season [28,53]. The eco-climatic model developed by Candeloro et al. [28], which is based on environmental covariates such as daytime and nighttime land surface temperature, normalised difference vegetation index, and surface soil moisture, is capable of generating risk maps for WNV spatial distribution probability throughout the Italian territory with 16 day-forecast periods (https://mapserver.izs.it/gis_wn_predictions/, accessed on 20 October 2022). In the 2022 WNV epidemic, it was able to indicate the presence of suitable conditions for an earlier (1–1.5 months) and wider spread of WNV in Italy (in particular, in Emilia-Romagna, Lombardy and Veneto regions (Results, Figure 6)). 

Despite the progress achieved in understanding the ecology and dynamics of West Nile virus in Italy, there are still significant knowledge gaps, especially on the role of different bird hosts as reservoirs and amplifiers of the infection and on the immune response of different species, which could affect the viral circulation among hosts and vectors. Therefore, new investigations to clarify the transmission and dynamics of viral spread are called for, as is the presence of overwintering phenomena that might allow a constant circulation of the virus from one season to the next. 

## 5. Conclusions

Our findings show that WNV L2 can persist in Italy, indicating an endemic circulation, which is probably sustained or amplified by several different reservoir species. Contrary to WNV L1, which mostly circulated in Italy between 2008 and 2011, and re-appeared on the Italian territory in the 2020–2022 epidemic seasons, WNV L2 has been constantly circulating in many regions of Italy. The substantial amount of WNV L2 data collected between 2011 and 2021 underline the fundamental importance of the Italian surveillance system in understanding the epidemiological scenario and in early detection of viral circulation (also in WNV-low risk areas and especially in regions presenting wild populations of birds subject to WNV infection). Additional epidemiological plans for active surveillance of animals received at rescue centres in Italy might also be important to promptly identify diseased birds, as suggested by Giglia et al. [9]. Deepening the knowledge on WNV ecology and transmission in wildlife would help the set-up of predictive epidemiological models for a better understanding of the viral dynamics in order to detect local spreads early on and to support the prompt implementation of response measures. 

## Data Availability

The data presented in this study, including sequence alignments and tree files, are openly available in FigShare at https://figshare.com/articles/dataset/WNV_L2_sequence_alignments/21518529, and https://figshare.com/articles/dataset/West_Nile_virus_lineage_2_tree_files/21518541/1, respectively, both accessed on 20 October 2022. All scripts used to perform this analysis are available at the https://github.com/andrea-silverj/WNV-L2_IT GitHub repository, accessed on 20 October 2022.

## Supplementary Materials

The following supporting information can be downloaded at: https://www.mdpi.com/article/10.3390/v15010035/s1, Figure S1: Molecular clocks of WNV genomes belonging to all known lineages; Figure S2: Phylogeography of WNV L2 in Italy for the seasons from 2010 to 2021; Figure S3: Phylogeography of WNV L2 in Italy for the seasons from 2010 to 2021; Figure S4: Coalescent Bayesian Skyline analysis of WNV L2 sequences; Table S1: Molecular and serological tests among bird, mosquitoes, horses, and humans; Table S2: Metadata of WNV sequences included in the study; Table S3: Dataset of models used; Video S1: Phylogeographic analysis of WNV L2 sequences_1; Video S2: Phylogeographic analysis of WNV L2 sequences_2; File S1: Group authorship list.
