# Supplementary material for: Epidemiological and Evolutionary Analysis of West Nile Virus Lineage 2 in Italy
Source: Viruses. 2022 Dec 22;15(1):35. doi: 10.3390/v15010035 (PMC9866873; doi:10.3390/v15010035)
Supplement: Supplementary file 1 [file viruses-15-00035-s001.zip › Supplementary_Figures.pdf]

Supplementary Figures

Supplementary Figure S1

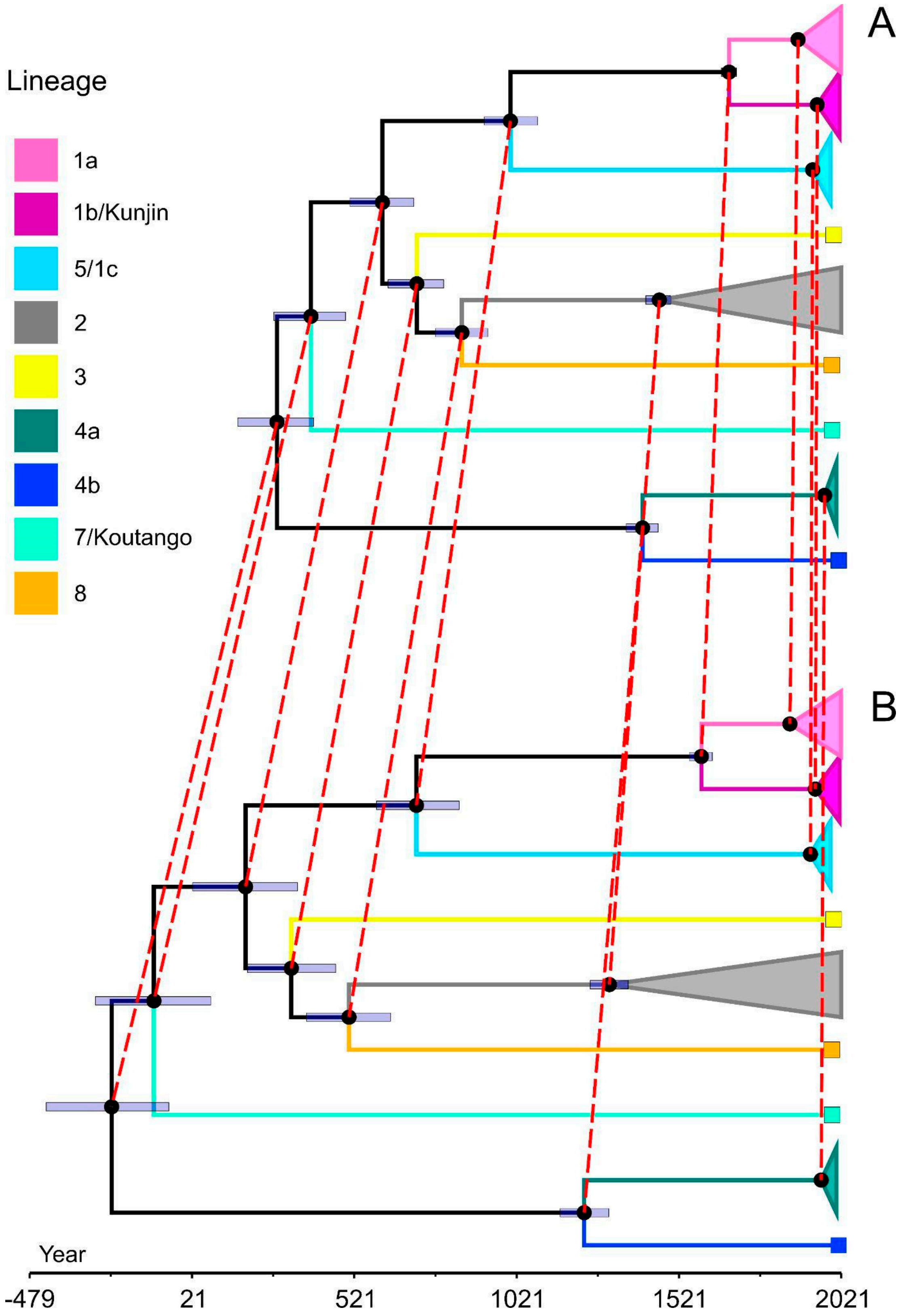

Supplementary Figure S2

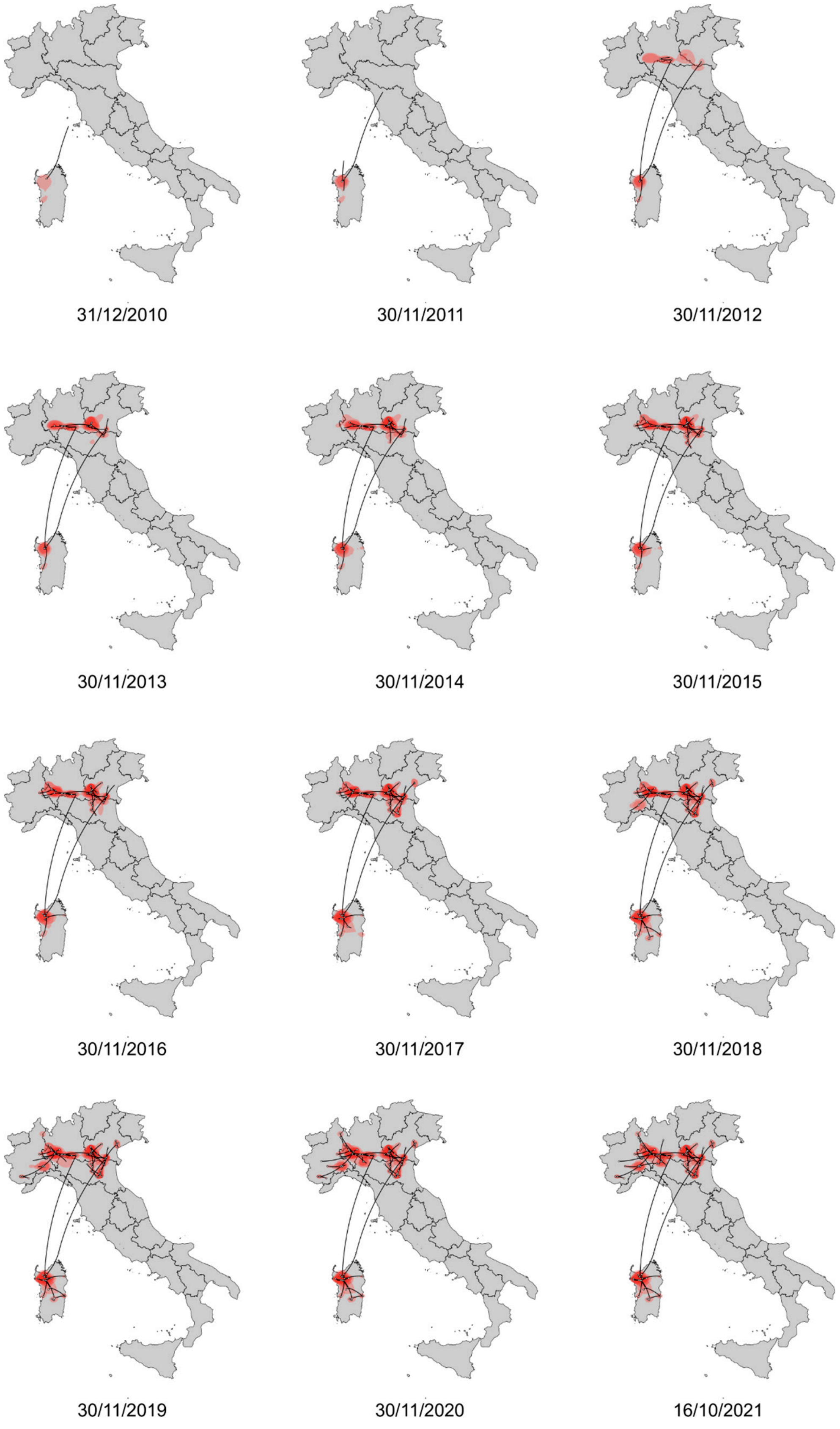

Supplementary Figure S3

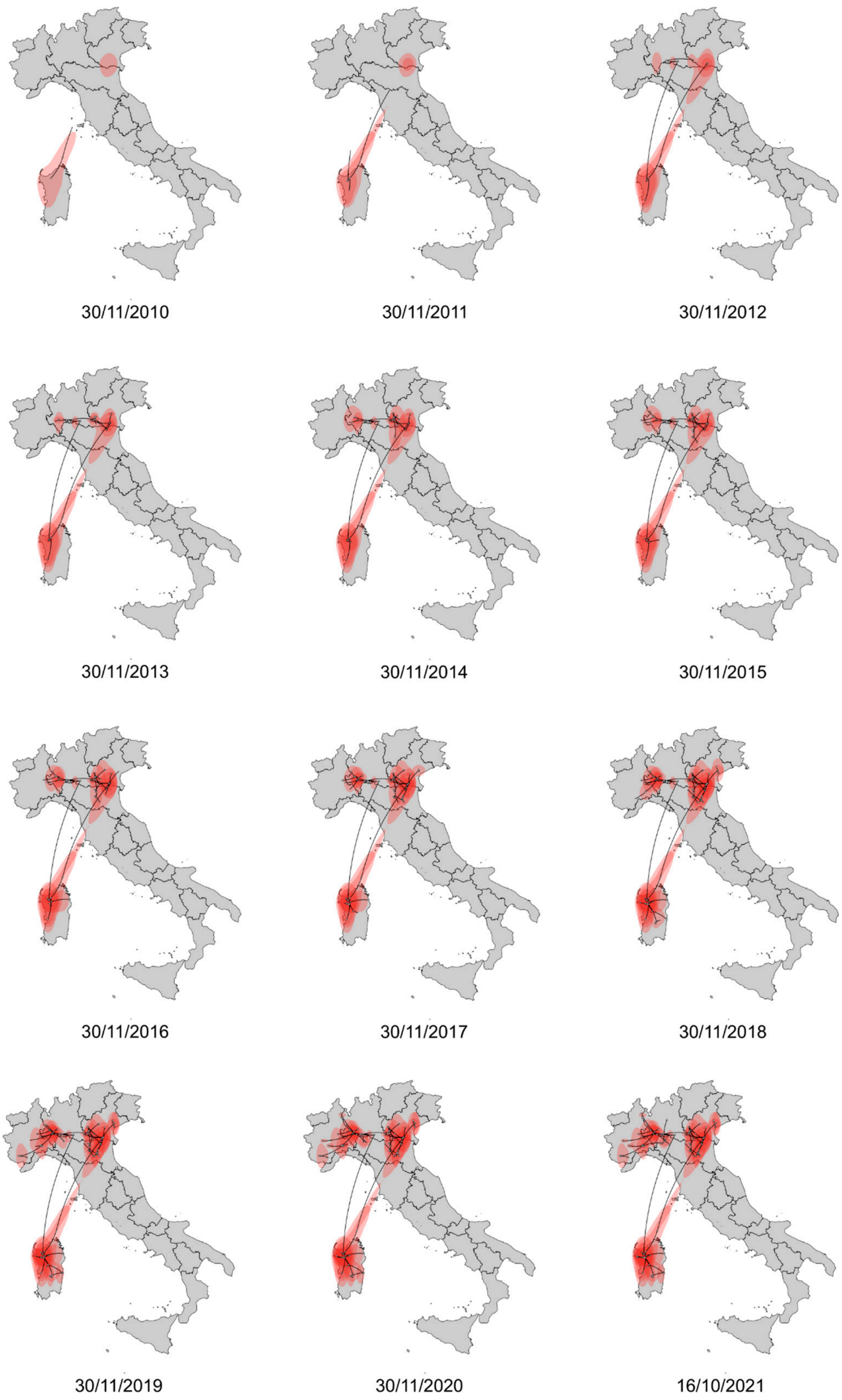

Supplementary Figure S4

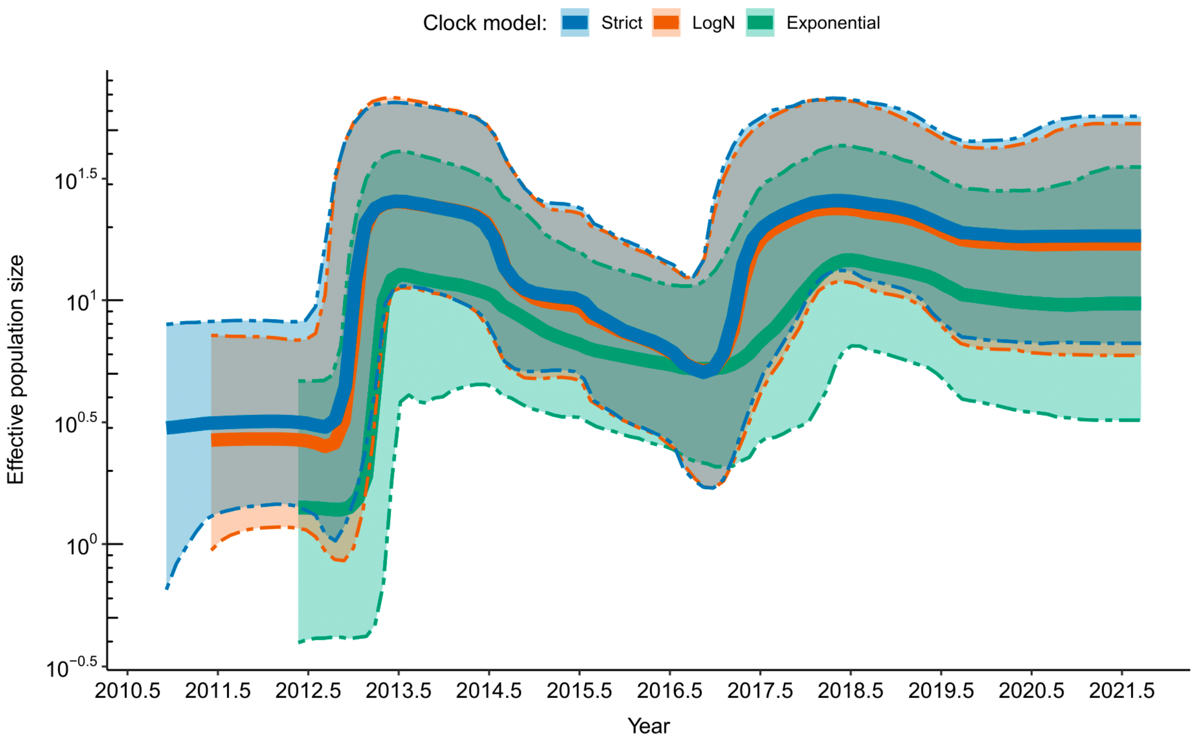

Supplementary figure captions

Supplementary Figure S1. Molecular clocks of 368 WNV genomes belonging to all known lineages. A) Coalescent-constant model. B) Birth-death model. The topology of the two trees is the same. The age estimates differ between the two models, with the birth-death tree systematically showing older dates. These differences are quite marked for deep nodes but become smaller for recent nodes.

Supplementary Figure S2. Phylogeography of WNV L2 in Italy for the seasons from 2010 to 2021.

Supplementary Figure S3. Phylogeography of WNV L2 in Italy for the seasons from 2010 to 2021, using the 95% HPDs.

Supplementary Figure S4. Coalescent Bayesian Skyline analysis of WNV L2 sequences, part of the identified Italian clade, reconstructed using 10 dimensions. Different clock models return similar results, showing an expansion of the population for the 2013 and 2018 epidemic seasons.
