# Supplementary figures and images for: Epidemiological and Evolutionary Analysis of West Nile Virus Lineage 2 in Italy
Source: Viruses. 2022 Dec 22;15(1):35. doi: 10.3390/v15010035 (PMC9866873; doi:10.3390/v15010035)

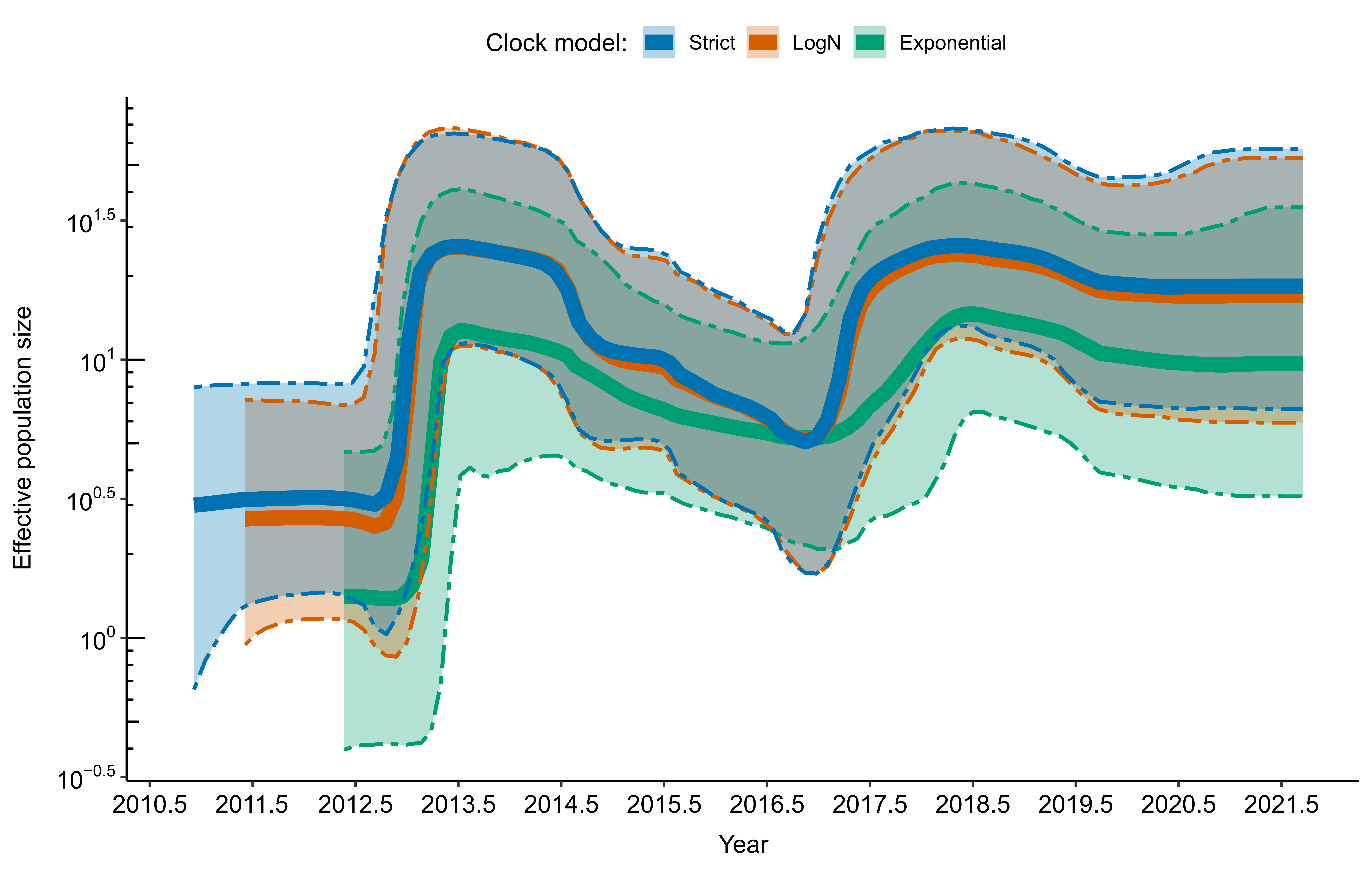

Supplement: Supplementary file 1 [file viruses-15-00035-s001.zip › SupplementaryFigure4.jpg]

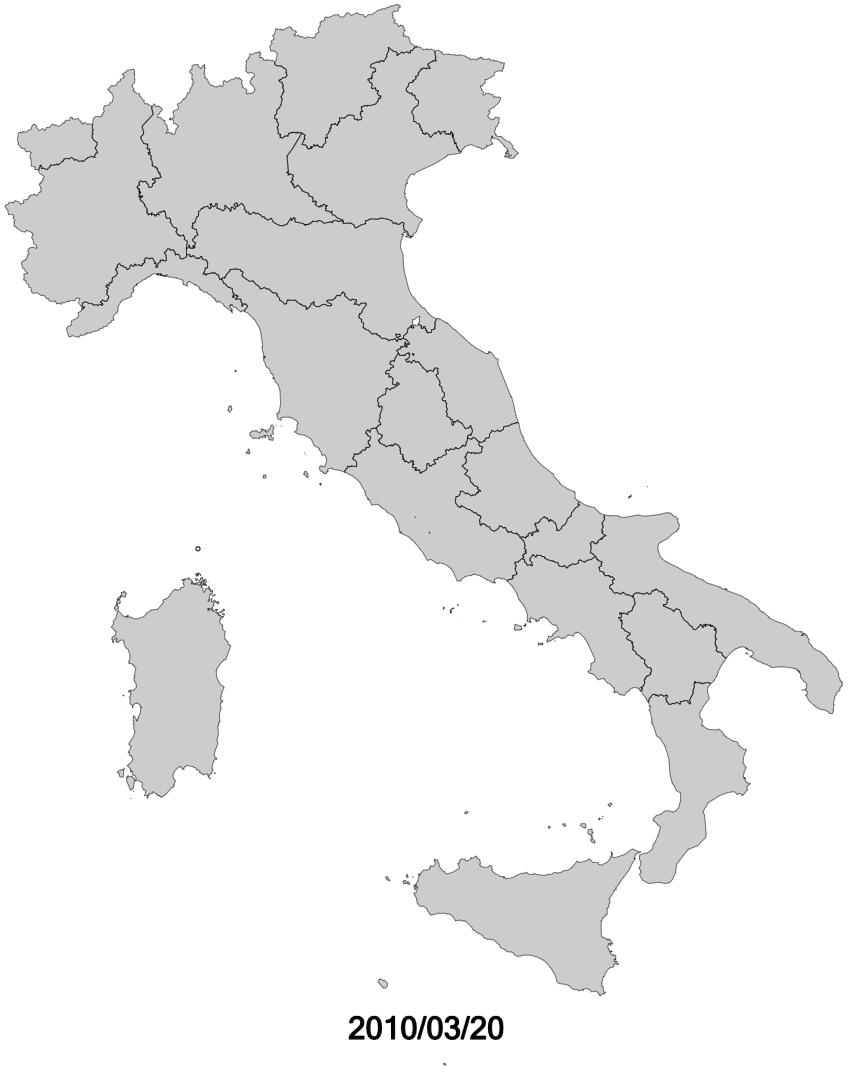

Supplement: Supplementary file 1 [file viruses-15-00035-s001.zip › Supplementary_Video_1.gif]

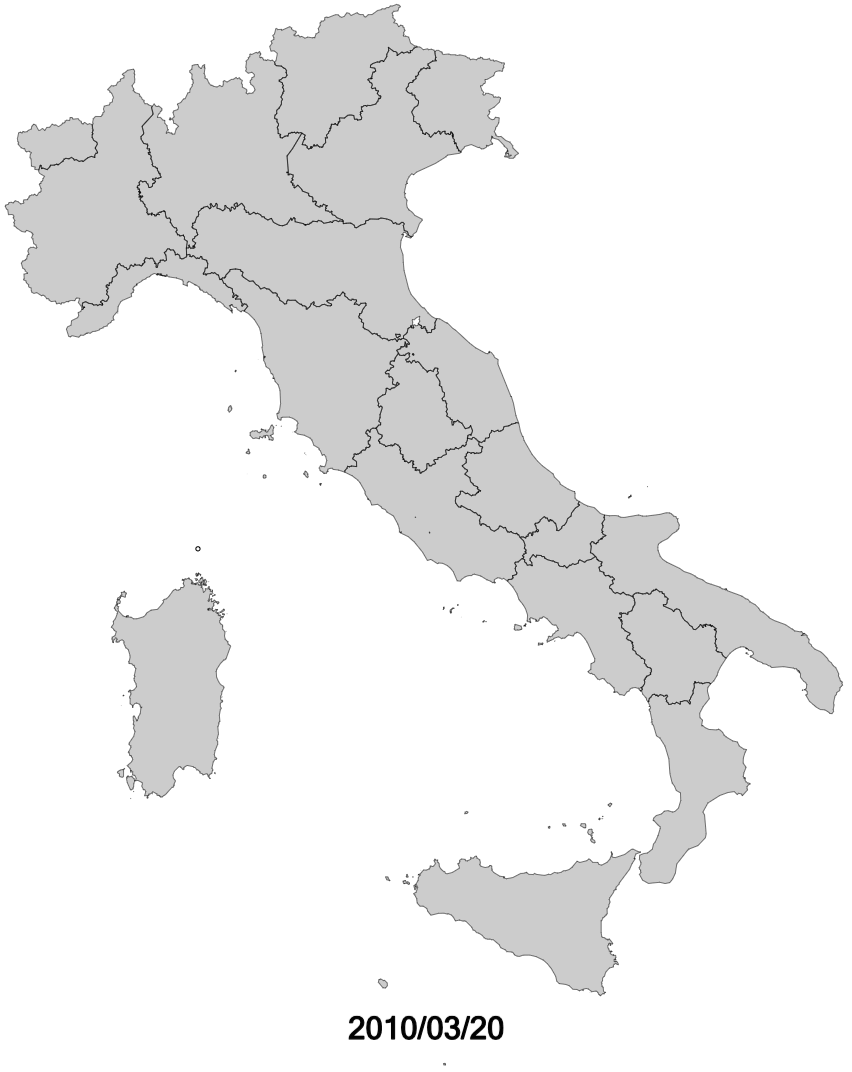

Supplement: Supplementary file 1 [file viruses-15-00035-s001.zip › Supplementary_Video_2.gif]
